# Supplementary figures and images for: The Influence of Nicotine on Trophoblast-Derived Exosomes in a Mouse Model of Pathogenic Preeclampsia
Source: Int J Mol Sci. 2023 Jul 5;24(13):11126. doi: 10.3390/ijms241311126 (PMC10342457; doi:10.3390/ijms241311126)

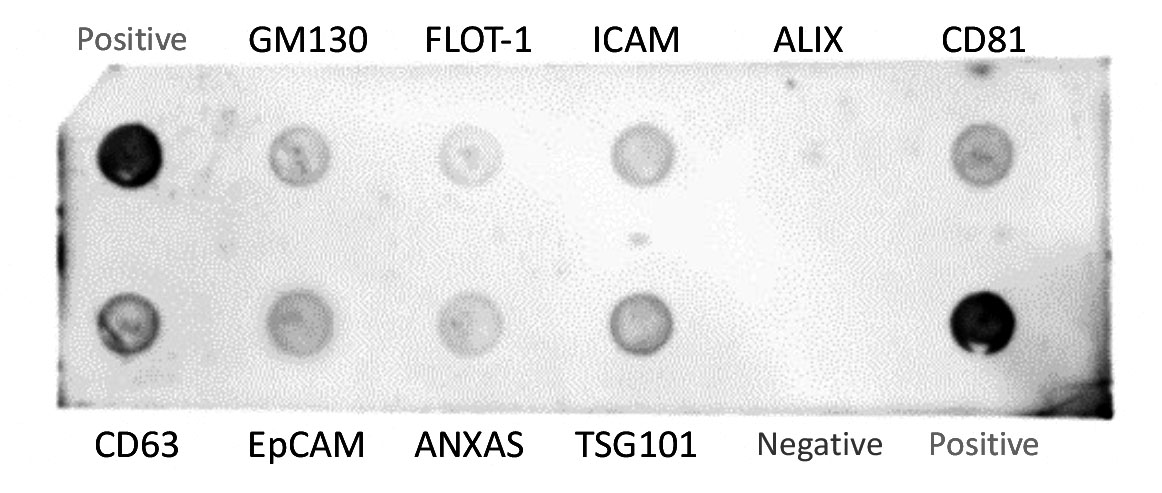

Supplement: Supplementary file 1 [file ijms-24-11126-s001.zip › ijms-2437772-supplementary.jpg]
